# Physicians’ Mutual Benefit Association

**Published:** 1888-03

**Authors:** 


### PHYSICIANS MUTUAL BENEFIT ASSOCIATION OF TEXAS.

Assessment No. 5 for benefit of certificate No. 25, held by Brother Dr. S. F. Starley, who died December 19, 1887, was sent to every member—157. Of these one hundred and forty paid, and seventeen forfeited their certificate for non-payment. One member resigned, after paying the assessment. This, with eight who have joined since Dr. Starley's death, leaves the membership 147 in good standing.

The $140 have been sent to Mrs. Starley, less cost of postal cards, the printing of same and postage on receipts, $4.50; and her receipt for $135.50 is published below:

Received of Drs. W. A. Morris President, and F. E. Daniel, Secretary Physicians' Mutual Benefit Association of Texas, One Hundred and Thirty-five Dollars.

(Signed) Mrs. S. F. Starley.

Tyler, Texas, March 24, 1888.

The following members paid assessment No. 5, death of Dr. S. F. Starley:

| | | |
|---|---|---|
| F. E. Daniel, | W. P. Burts, | A. S. Wolff, |
| S. W. Sholars, | Geo. Cupples, | F. Herff, |
| D. V. Spring, | R. M. Swearingen, | R. H. Day, |

J. B. Duchiene,
J. P. Coffey,
F. T. Paine,
R. G. Williams,
W. M. Powell,
L. H. Hardy,
J. W. Daniel,
A. D. Paulus,
G. W. Gray,
E. S. Tidwell,
T. J. Bennett,
H. H. Thorpe,
J. R. Scales,
T. C. Osborn,
T. J. Murph,
J. M. Litten,
W. G. Jameson,
W. T. Richmond,
J. H. Evans,
J. T. Field,
J. W. Fennell,
J. H. Bowers,
Jos. Greer,
W. H. Lancaster,
W. J. Dupree,
T. J. Watlington,
S. A. Stone,
M. F. Wakefield,
J. M. Fry,
C. Richardson,
J. A. Southworth.
J. G. Daniels,
A. W. Crawford,
J. W. Hamilton,
W. A. Taylor,
J. G. Barbee,
G. J. Starnes.
J. D. Beck,
J. W. Dupree,
T. M. Matthews,
H. W. Brown,
S. F. Styles,
M. A. Taylor,
C. O. Weller,
Jno. Inabnit,
J. T. Webb,
J. M. Ball,
A. M. Hill,
J. M. Ross,
J. A. Throckmorton,
A. N. Perkins,
J. E. Mayfield,
Q. C. Smith,
J. H. McClintock,
F. R. Martin,
J. M. Lewis,
J. T. Y. Jamison,
A. Wadgymar,
Q. A. Shuford,
A. L. Patton,
M. R. Denman,
C. M. Harrison,
C. Sams,
T. G. May,
T. J. Foster,
R. A. Taylor.
A. D. Evans,
T. J. Bell,
H. F. Witherspoon,
J. D. Bass,
Z. T. Bundy,
W. M. Burger.
G. W. Cain,
C. Bullitt,
J. F. W. Metzler,
H. M. Burroughs,
S. A. Towsey,
M. D. Sterrett,
W. H. Wilkes,
M. M. Myers,
J. W. McLaughlin,
T. F. McFarland,
F. A. Tompkins,
Y. D. Harrington,
R. W. Reid,
W. A. Morris,
C. C. Francis,
J. M. Hons,
J. R. Keating,
F. C. Ford,
A. D. Tinsley,
Sam J. Fox,
R. S. Gregg,
T. M. Wilson
J. K. Fraser,
W. Glover,
T. S. Burke,
A. Patton,
R. P. Talley,
J. A. Denson,
J. H. T. King,
J. Getzwiller,
W. A. McGee,
J. B. Stinson,
F. W. Kaiser,
E. R. W. McCreary,
E. Jones,
J. T. Musick,
R. P. Davis,
T. W. Styles,
A. N. Denton,
E. G. Nicholson,
J. A. Abney,
L. D. Hill,

| | | |
|---|---|---|
| D. M. Vawter, | R. H. Harrison, Sr., | R. H. Harrison, Jr., |
| A. McDaniel, | Todd Robinson, | L. T. Maddox, |
| J. S. Bruce, | Jessee E. Grace, | H. C. Grace, |
| W. B. Treadwell, | Jack Phillips, | Bat Smith, |
| Jno. K. Davidson, | J. T. Phillips, | E. P. Becton, |
| W. T. Strain, | | F. A. Maxwell. |

| | | |
|---|---|---|
| Total enrollment to date | | 217 |
| Forfeited previously | 47 | |
| Died | 5 | |
| Resigned | 1— | 53 |
| | | 164 |
| Forfeited on assessments No. 5 and A. D. | | 17 |
| Number in good standing | | 147 |

By comparing the list above, with the publication in the January number of those who paid assessment No. 5, the names of those who failed to pay No. 4, can be ascertained, we do not feel authorized to indicate them otherwise.

